# Endoscopic Versus Microscopic Type 1 Tympanoplasty (Myringoplasty) in a Rural Tertiary Care Hospital in India: A Retrospective Comparative Study

**DOI:** 10.7759/cureus.36109

**Published:** 2023-03-13

**Authors:** Megha Kawale, Suhas Landge, Deepika Garg, Kashyap Kanani

**Affiliations:** 1 Department of Otorhinolaryngology, Mahatma Gandhi Institute of Medical Sciences, Wardha, IND; 2 Department of Orthopedics, Jawaharlal Nehru Medical College, Datta Meghe Institute of Higher Education and Research, Wardha, IND

**Keywords:** air-bone gap, chronic otitis media, microscopic tympanoplasty, endoscopic ear surgery, tympanoplasty

## Abstract

Background

Chronic suppurative otitis media (CSOM) is described as middle ear cleft inflammation that results in long-term alterations to the tympanic membrane and/or the middle ear structures. In cases of CSOM, type 1 tympanoplasty, also known as myringoplasty, is a successful procedure for repairing the tympanic membrane and can even help restore hearing loss. This study aims to compare functional and clinical outcomes of type 1 tympanoplasty performed using transcanal endoscopic ear surgery (TEES) versus those performed via microscopic ear surgery (MES) for perforation in the tympanic membrane in the safe type of CSOM.

Methodology

Between January 2018 and January 2022, a retrospective analysis of 100 patients (47 men and 53 women) operated for the safe type of CSOM with a perforated tympanic membrane was conducted in our department. Based on the surgical methods, cases were randomly divided into two groups. There were 50 people in group 1 who underwent endoscopic tympanoplasty and 50 in group 2 who underwent microscopic tympanoplasty. The following factors were assessed: patient demographics; tympanic membrane perforation size at the time of surgery; operating room time; hearing outcomes, that is, closure of air-bone gap (ABG); graft uptake success rate; postoperative hospital stay; and medical resource usage. Patients were followed up for 12 weeks.

Results

Both groups shared similar epidemiological profiles, preoperative hearing status, and perforation sizes. In both groups, the rate of graft uptake was comparable. The average ABG closure was also quite comparable. In the case of endoscopic surgeries, the mean operative time was shorter; which was statistically significant, and complications were significantly lower in group 1.

Conclusions

Compared to its microscopic counterpart, endoscopic tympanoplasty has a similar graft uptake success rate and a comparable hearing outcome; however, it requires less operative time and hospital stay, has early recovery, and makes lesser use of medical resources, and it is cosmetically better.

## Introduction

Chronic suppurative otitis media (CSOM) is described as the inflammation of the middle ear cleft that results in long-term alterations to the tympanic membrane and/or the middle ear structures. It is further divided into cholesteatomatous and noncholesteatomatous [1].

In cases of CSOM, type 1 tympanoplasty, also known as myringoplasty, is a successful procedure for repairing the tympanic membrane and can even help restore hearing loss [2]. Type 1 tympanoplasty consists solely of repairing tympanic membrane perforations. Berhold performed it for the first time in 1878, and later in 1950, Wullstein and Zollner made it popular [3]. Ever since, numerous techniques have been developed for reconstructing the tympanic membrane. The preferred graft materials included cartilage, perichondrium, canal wall skin, and veins, although temporalis fascia is still by far the most widely used graft material [4]. Worldwide, it is frequently carried out by microscopic ear surgery (MES) [5]. In 1921, Swedish otolaryngologist Carl Olof Nylen used a monocular microscope for the first time. Later, in 1922, Gunner Holmgren used a binocular microscope. However, those microscopes were not used widely because of unstable illumination, poor light quality, a small field of vision, and a very close focal distance. All previous models were replaced in 1951 by a new, improved model created by the Littman and Zeiss company [6]. The modern microscope has the benefit of binocular vision, superb stereoscopic surgical vision, good depth perception, and frees both hands, but it also has the drawback of straight-line vision, which makes it challenging to see the numerous deep recesses in the middle ear [7]. A new technology called the endoscopic camera was developed to overcome this drawback. Several surgeons have successfully substituted endoscopes for microscopes for partial ear surgery since the late 1900s [8]. Trans-canal endoscopic ear surgery enables wide-angle vision, a cone-shaped light source that ensures optimal visualization, magnification of the middle ear structures, and direct visualization of obscure areas like the hypotympanum, sinus tympani, epitympanum, and posterior part of the mesotympanum [9]. In modern otosurgery, the endoscope plays an important role. With an endoscope, even cochlear implant surgery is possible that too with more precision and even in challenging anatomical conditions such as ear deformities and progressive otosclerosis. The endoscope makes it easier to identify the round window area, thereby making it effective, easier, and safe implantation. It is possible to avoid posterior tympanotomy and reduce the chance of facial nerve damage during endoscopically assisted cochlear implant procedures [10].

A few studies have compared the results of microscopic and endoscopic tympanoplasty, such as the percentage of graft uptake, intraoperative procedure time, postoperative hospital stay, and improved hearing. In this study, we aim to compare various aspects (functional and clinical outcomes) of both procedures and determine the benefits and drawbacks of one over the other.

## Materials and methods

This study was performed as a retrospective comparative study of 121 patients at Mahatma Gandhi Institute of Medical Sciences who underwent type 1 tympanoplasty from January 2018 to January 2022. Out of 121 patients, 100 patients were included in the present study and 21 patients were excluded due to the presence of intraoperative cholesteatoma, ossicular chain defect, and tympanic membrane retraction pocket, resulting in the application of different correction surgeries.

The patients were randomly divided into the following groups: group 1 - endoscopic tympanoplasty; group 2 - conventional microscopic tympanoplasty.

All patients included in the study had dry ears for six weeks, and we used a temporalis fascia graft with an underlay technique in all the surgeries. Demographic data, preoperative size of tympanic membrane perforation, time for surgery, graft uptake success rate, and postoperative hearing assessment were documented from the available database.

Exclusion criterion

Patients aged below 16 years and above 60 years and with CSOM complications, narrow external auditory canal (EAC), added sensorineural hearing loss (SNHL), and cholesteatoma were excluded.

Audiological assessment

Pre- and postoperative hearing assessments were done, and each patient's ABG was evaluated by averaging their hearing threshold at 0.5, 1, 2, and 4 kHz.

Anesthesia

In our hospital, most of the microscopic tympanoplasties were performed in general anesthesia (GA), while all the endoscopic procedures were performed in local anesthesia (LA) as the duration of the former is greater than the latter. However, endoscopic procedures can also be performed under GA according to the patient's preference or willingness.

Surgical techniques

**Figure 1:**
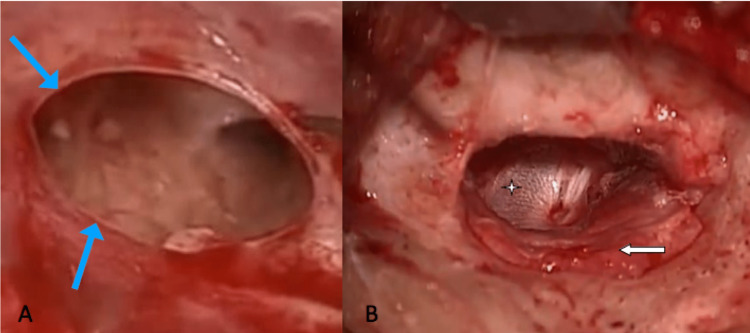
(A) Microscopic preoperative appearance of a perforation in the left tympanic membrane (blue arrows); (B) microscopic appearance of temporalis fascia in situ (star) with reposed tympanomeatal flap (white arrow).

In our Institute, the postauricular approach was used for all conventional microscopic tympanoplasties to obtain a wider surgical view. In this procedure, graft tissue of temporalis fascia was harvested via post auricular Wilde's incision followed by the freshening of the edges of perforation (Figure 1A) and creation of vascular strip by giving six o'clock and 12 o'clock incision followed by access to the tympanic cavity via elevation of the tympanomeatal flap (TM flap). All the inflamed and infected tissue in the tympanic cavity was then debrided thoroughly. The ossicular chain was assessed intraoperatively before placing the graft tissue on the undersurface of the TM flap to reconstruct the tympanic membrane (Figure 1B). Finally, the middle and external ear canals are packed with an absorbable gelatin sponge.

**Figure 2:**
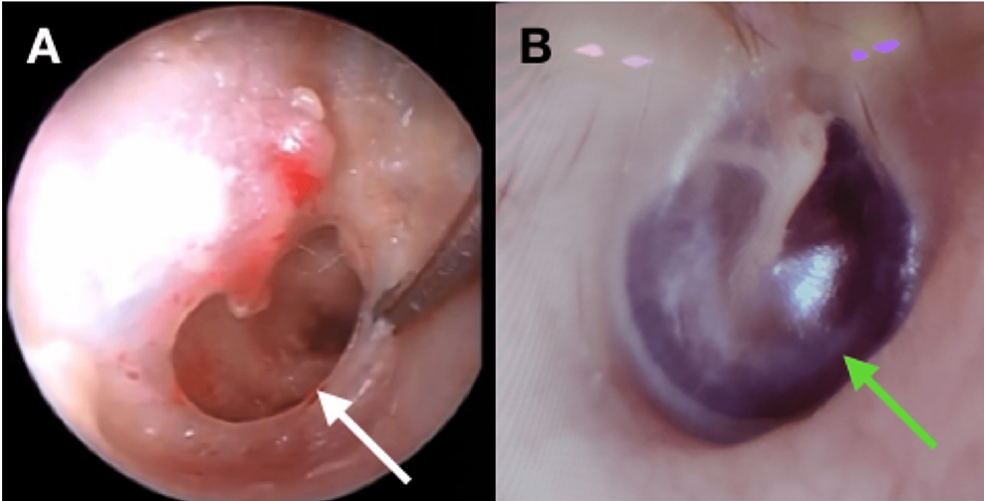
(A) Preoperative endoscopic appearance of a perforated tympanic membrane (white arrow); and (B) endoscopic appearance of the healed tympanic membrane (green arrow).

For endoscopic tympanoplasty, the patients were given intramuscular premedication half an hour before the surgery in the form of injection of Pentazocine 30 mg, injection of Promethazine 25 mg, and atropine 0.4 mg to sedate, prevent preoperative vomiting, and counter any vagal oversensitivity, respectively. LA (xylocaine 1% with 1:100,000 adrenalin) was administered in the postauricular and four quadrants of the EAC, and temporalis fascia was harvested above the hairline using a supra auricular incision. Rigid endoscopes (Karl Storz Endoscopes, Tuttlingen, Germany) of 30° and 0° angles and having diameters of 2.7 and 11 cm, respectively, were used. Through a high-definition (HD) camera images were transferred to the HD monitor. With the help of an endoscope, the edges of the perforation were freshened (Figure 2A), the endoscope was passed through the perforation into the tympanic cavity, and the ossicles were assessed. Using a circular knife, a canal wall incision was given from 12 o'clock to six o'clock in the posterior canal wall 7 mm lateral to the annulus, and an anteriorly based TM flap was elevated. Ossicular chain integrity was confirmed before placing gel foaming in the middle ear. A temporalis fascia graft of adequate size was then placed by the underlay method, and the TM flap was reposed over the graft. In the end, the meatus was packed with an absorbable gelatin sponge soaked in an antibiotic solution, and a small dressing was applied.

Outcome measures

Surgical outcomes, restoration of hearing, and use of medical resources were the main result components. Within two weeks, the external ear packing was removed. Then the patients were followed up every two weeks until complete recovery. The graft uptake assessment and a pure tone audiogram (PTA) were performed 12 weeks after surgery (Figure 2B).

Evaluation of outcome

All available medical records were reviewed retrospectively, and independent reviewers collected the data who were not involved in surgeries or postoperative follow-up to diminish observer bias. All the surgeries were performed by a senior surgeon (the first author). Patients were divided into two groups.

Four results were examined and compared between the groups: (1) the results of the surgery, including the effective tympanic membrane healing, graft uptake, and any postoperative problems; (2) restoration of hearing abilities; (3) use of healthcare resources; and (4) the average amount of time (in minutes) spent in the operating room, including the time spent for anesthesia administered.

Statistical analysis

Descriptive and inferential statistics were used for statistical analysis using the chi-square test and student's paired and unpaired t-tests. Analysis was done by SPSS (27.0 version, IBM Corp., Armonk, NY, USA) and GraphPad Prism 7.0 version software (GraphPad Software Inc., San Diego, CA, USA), and *P *< 0.05 was considered as the level of significance.

## Results

 

Our series included 100 patients (47 males [47%] and 53 females [53%]) with age ranges between 16 and 60 years, with a mean age of 30.51 ± 10.42 years. In group 1, there were 30 males and 20 females who were operated on endoscopically, whereas in group 2, there were 17 males and 33 females who were operated on microscopically. Endoscopic tympanoplasty was performed on 20 right and 30 left ears, and microscopic tympanoplasty was performed on 22 right and 28 left ears (Table 1). For the endoscopic procedure, the operative time (range 30-70 minutes) was 49.22 ± 8.24 minutes (mean ± standard deviation [SD]), and for MES, it (range 80-110 minutes) was 83.20 ± 14.73 minutes. The mean operative time difference was statistically significant (*t *= 31.24), with *P *< 0.0001 (Table 1). The graft uptake rate at 12 weeks in the case of endoscopic tympanoplasty was 80% (40 grafts in situ), which was comparable to that of MES - 78% (39 grafts in situ). The difference was not statistically significant (Table 1).

**Table 1:** The demographic characteristics and surgical outcomes of the patients. ϗ^2^-value, chi-square value; *P*-value, probability value; *t*-value, Student's *t*-test value; S, significant; NS, nonsignificant

	Endoscopic group (*N* = 50)	Microscopic group (*N* = 50)	Test of significance
Gender, *n* (%)			
Male	30 (60)	17 (34)	
Female	20 (40)	33 (66)	
Mean age (range in years)	29.68 ± 10.56 (16-55)	31.34 ± 10.31 (16-53)	
Side			
Right	20	22	
Left	30	28	
Perforation size, *n* (%)			*ϗ*^2^-value = 1.50; *P*-value = 0.47; NS; *P *> 0.05
Large	23 (46)	26 (52)	
Medium	14 (28)	16 (32)	
Small	13 (26)	8 (16)	
Graft uptake rate (%)	40 (80)	39 (78)	ϗ^2^-value = 15.08; *P*-value = 0.0001; S; *P* < 0.05
Mean operative duration (range)	49.22 ± 8.24 minutes (30-70 minutes)	83.20 ± 14.73 minutes (80-110 minutes)	*t*-value = 31.24; *P*-value = 0.0001; S

 

Comparison of hearing outcomes

In our study, preoperative PTA was done in both groups and postoperative PTA was done 12 weeks after surgery. Postoperative PTA improvement was assessed after 12 weeks since surgery in both study groups. The mean hearing in the endoscopic surgery group was 29.10 dB, while in the microscopic surgery group, it was 29.20 dB (Table 2).

 

 

**Table 2:** Postoperative PTA improvement in both study groups (independent t-test for surgery outcome). PTA, pure tone audiometry; *P*, probability value; NS, nonsignificant; *N*, number of patients; *t*-value, Student's *t*-test value

Group	N	Mean	Standard deviation	Standard error mean	*t*-value
Endoscopic surgery	50	29.10	2.70	0.38	0.16, *P *= 0.84, NS
Microscopic surgery	50	29.20	3.34	0.47	

Hearing improvement before and after surgery shows significant improvement in both the endoscopic and microscopic surgery groups. In the endoscopic surgery group before surgery, mean hearing improved from 39.66 to 29.10 dB, while in the microscopic surgery group, hearing improved from 39.82 to 29.20 dB (Table 3).

**Table 3:** Hearing improvement after the surgery paired t-test for each type of surgery (descriptive statistics). *N*, number of patients

		Mean	N	Standard deviation	Standard error mean
Pair 1	Before endoscopic surgery	39.66	50	4.34	0.61
	After endoscopic surgery	29.10	50	2.70	0.38
Pair 2	Before microscopic surgery	39.82	50	3.97	0.56
	After microscopic surgery	29.20	50	3.34	0.47

The ABG closure was 10.56 dB in the endoscopic group and 10.67 dB in the microscopic group but was statistically insignificant. The independent *t*-test value for the surgical outcome was 22.23 for the endoscopic surgery group and 20.53 for the microscopic surgery group, and the *P*-value was 0.0001 in both groups, which was very significant, indicating that there was no statistically significant variation in the outcome depending on the type of operation. Equally significant hearing improvement was observed in both groups (Table 4).

**Table 4:** Hearing improvement after the surgery paired t-test for each type of surgery (paired samples test). df, degrees of freedom; *P*-value, probability value; *t*-value, Student's *t*-test value; S, significant

		Paired differences					*t-*value	df	*P*-value
		Mean	Standard deviation	Standard error mean	95% confidence interval of the difference				
					Lower	Upper			
Pair 1	Endoscopic surgery	10.56	3.35	0.47	9.60	11.51	22.23	49	0.0001, S
Pair 2	Microscopic surgery	10.62	3.66	0.51	9.58	11.66	20.53	49	0.0001, S

We also compared the incidents of complications in both groups. Group 1 encountered complications such as severe SNHL > 70 dB in one patient (2%) and otitis externa in three patients (6%), whereas group 2 encountered mastoiditis in one patient (2%), persistent otorrhea in two patients (4%), and wound dehiscence in two patients (4%; Table 5).

**Table 5:** Complications of endoscopic tympanoplasty and microscopic tympanoplasty. SNHL, sensorineural hearing loss

Complications	Endoscopic surgery, *n* (%)	Microscopic surgery, *n* (%)
Severe SNHL	1 (2)	
Mastoiditis		1 (2)
Persistent otorrhea		1 (2)
Wound dehiscence		2 (4)
Otitis externa	3 (6)	

Postoperative hospital stay

**Figure 3:**
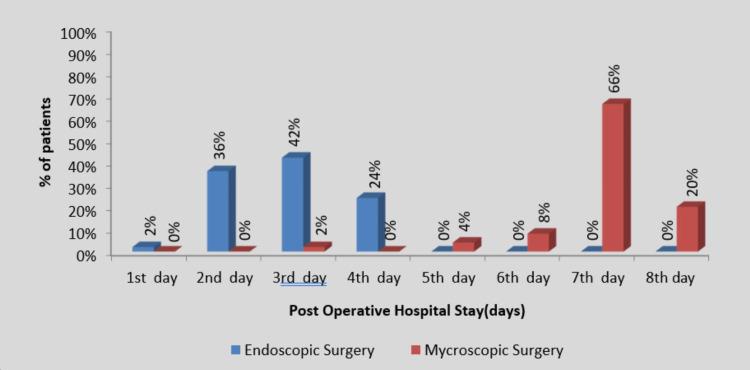
Postoperative hospital stay in endoscopic tympanoplasty and microscopic tympanoplasty.

Most of the patients from group 1 were discharged on postoperative days 2 and 3. Those in group 2 were mostly discharged on postoperative days 7 and 8, so overall, ϗ^2^ values = 96.18 and *P*-value = 0.0001 (*P *< 0.05) were statistically significant. It means the endoscopic tympanoplasty group requires less postoperative hospital stay as compared to the microscopic tympanoplasty group (Figure 3).

 

## Discussion

Tympanoplasty is a therapy option for CSOM that primarily aims to heal the infection, fix the ruptured tympanic membrane, and enhance hearing [11]. With the benefit of two-handed manipulation, binocular vision, and an excellent stereoscopic surgical view, traditional MES using an endaural or postauricular approach is associated with a success rate of 83% to 100% [12-14]. In a postauricular method, soft tissue retraction, with or without drilling of bone, is required to accomplish the same results because surgeons cannot detect concealed areas such as the anterior boundary of the tympanic membrane, sinus tympani, or facial recess with the straight-line vision of microscopes [15,16]. This could lengthen the procedure, make general anesthesia necessary, and raise the likelihood of postoperative complications [16,17]. In our study, we found that the average operation time of group 2 was significantly longer than that of group 1 (*t *= 31.24; *P *= 0.0001) because the former required more soft tissue dissection, bone drilling (in a few cases), and a lengthy period to close the wide wound, whereas, in the latter, endoscopes offered a superior surgical view, required a smaller surgical incision, and preserved more patient tissue because they used a transcanal approach. Additionally, only some patients in group 2 had general anesthesia, whereas all of the patients in group 1 received local anesthesia.

In group 1, patients were typically discharged on the first and second postoperative days, but in group 2, patients were released on the seventh and eighth postoperative days, resulting in a shorter postoperative recovery period and hospital stay. Additionally, compared to endoscopic ear surgery, microscopic tympanoplasty necessitated spending more time overall in the operating room and more medical resources. This finding is in line with earlier research, such as that by Hsu et al. [18], who found that endoscopic tympanoplasty required less time during surgery than microscopic tympanoplasty and that postoperative problems were less frequent in the former. According to a study by Kozin et al. [16], endoscopic ear surgery has definite advantages. The external auditory canal is converted into a surgical portal, reducing the time needed to complete the procedure, which can be done comfortably under local anesthesia. This minimally invasive transcanal approach also causes less damage to soft tissues and eliminates the need for a postauricular incision and canaloplasty. In addition to enhanced cosmesis, it was linked to a quicker postoperative recovery, measured by an early return to regular daily activity and an early discharge of patients [19].

At the end of 12 weeks, the graft uptake rates in the transcanal endoscopic (80%) and microscopic (78%) tympanoplasty groups were comparable. In the endoscopic ear surgery group, one patient had severe SNHL as a postoperative complication, three had otitis externa, and one had mastoiditis. In the MES group, one patient had persistent otorrhea, or discharge lasting more than two months, and two had postauricular wound dehiscence. Overall, there was no statistically significant difference in the outcomes of the two surgeries. According to the literature review and meta-analysis study by Tseng et al. [20], graft uptake success rates for endoscopic and microscopic tympanoplasty, respectively, were 85.1% and 86.4%, with no discernible difference. The same was verified by Choi et al. [21] in a separate trial, who found graft uptake success rates of 100% and 95.8% in the endoscopic and microscopic groups, respectively (*P *= 0.304). For the microscopic group, the mean operative time was 88.99 ± 28.5 minutes, but for the endoscopic group, it was 68.2 ± 22.1 minutes (*P* = 0.002). Thus, it was evident that the surgical time had been significantly reduced due to the use of endoscopes, corroborating the findings of our study.

In 2015, a study by Mokbel et al. [22] found that the graft uptake rate was 100% in the endoscopic group and 90% in the microscopic group, with follow-up extending from six to 24 months. The endoscopic group's surgical time was 40 ± 5.50 minutes, whereas the microscopic group's surgical time was 55 ± 10.50 minutes (*P *= 0.0001).

An improvement in the hearing was a key factor in determining the surgical outcome of the treatment. According to a study by Hsu et al., there were no statistically significant differences between the transcanal endoscopic ear surgery (TEES) and MES groups in hearing restoration, including pre- and postoperative ABG, average hearing gain (in decibels) (10.3 ± 6.4 for TEES and 12.4 ± 7.5 for MES, *P* = 0.1663) and the percentage of patients with improved hearing after surgery (TEES versus MES: 96.2% versus 94.0%, *P* = 0.4848). This implies that TEES shows similar results to MES for the restoration of hearing after surgery. Regarding hearing restoration, we found no statistically significant difference between the two procedures (10.56 ± 3.35 for endoscopic versus 10.62 ± 3.66 for microscopic, *P *= 0.0001). Another study conducted by Dündar et al. [23] also produced comparable outcomes, with a postoperative hearing gain of 20.40 dB against 21.34 dB (*P *> 0.05) and postoperative ABG of 8.12 dB versus 8.13 dB. *P* > 0.05 in both endoscopic and microscopic procedures.

In our study, we were able to achieve an 80% graft success rate following endoscopic tympanoplasty with minimal postoperative complications, satisfactory improvement in hearing, shorter hospital stays, faster recovery, lower financial burden, and earlier restoration of normal daily activity, which is more crucial for rural areas in India where the majority of the population is engaged in agriculture and employed daily. Therefore, we think that endoscopic ear surgery is a good alternative to microscopic tympanoplasty because it produces positive surgical and postoperative results.

There are several limitations of this retrospective study. We did not include other graft materials like tragal cartilage in our study as the data was incomplete and less. Second, other approaches, such as the endaural approach, are not included in the study as a senior surgeon in our hospital usually does it. As this is a single-center study in a rural hospital where living conditions are poor and the rate of infection is more than in the urban and educated populations, so results of the study may not be applicable worldwide. A more extensive survey of multihospital cases with relatively long-term follow-up results would be beneficial.

## Conclusions

Endoscopic transcanal ear surgery in type 1 tympanoplasty follows the same basic principle as microscopic surgery, and graft uptake rate and hearing improvement are comparable. Without an external incision or EAC drilling, the edge of the perforation cannot be easily reached by microscopic tympanoplasty. As an endoscopic technique has a wider field of view than a microscopic treatment, less surgical tissue damage is required to provide adequate operating exposure. Patients who undergo endoscopic tympanoplasty experience less canaloplasty and more acceptable cosmetic outcomes. In addition, endoscopic tympanoplasty takes less time for surgery, has a shorter hospital stay and earlier postoperative recovery, and uses lesser medical resources, making it an excellent alternative to the traditional microscopic approach.
